# GutMGene-Guided Peripheral Blood Transcriptomics Identifies an FLNA-Associated Host-Gene Signal in Diabetic Retinopathy

**DOI:** 10.3390/ijms27146182

**Published:** 2026-07-10

**Authors:** Chuanxue Ma, Yujun Wang, Yi Liu

**Affiliations:** Nanjing Hospital of Chinese Medicine, Nanjing University of Chinese Medicine, Nanjing 210000, China; chuanxue2019@163.com (C.M.); 202511551@njucm.edu.cn (Y.W.)

**Keywords:** diabetic retinopathy, gutMGene, *FLNA*, peripheral blood transcriptomics, WGCNA, scTenifoldKnk, virtual knockout, immune–vascular interactions

## Abstract

Diabetic retinopathy (DR) reflects retinal microvascular injury and systemic immune-metabolic stress, and most public DR transcriptomic datasets lack paired microbiome/metabolomic profiles. We used gutMGene v2.0 as a curated microbe/metabolite–host gene prior and integrated it with peripheral blood transcriptomics from GSE221521. Candidate genes were refined by weighted gene co-expression network analysis (WGCNA), repeated resampling, cross-dataset assessment, mechanism scoring, peripheral blood mononuclear cell (PBMC) single-cell localization and filamin A (*FLNA*)-centered single-cell gene regulatory network (GRN) virtual knockout. The gutMGene prior contained 238 host genes; 15 DR-associated genes overlapped this prior, and WGCNA retained ten candidate gut microbe and microbial metabolite-related genes (GMMRGs): *FLNA*, *AKT1*, *IRAK1*, *BCL10*, *CDK6*, *CTSD*, *JUP*, *CXCL1*, *CXCR2* and *IL4R*. Resampling prioritized *FLNA* as the most consistent candidate. Cross-dataset assessment localized the strongest signal to type 2 diabetes (T2D) PBMCs, retinal endothelial cells and advanced proliferative diabetic retinopathy with diabetic macular edema (PDR + DME) retinal tissue, with weaker separation in whole blood, broad retinal tissue and six-donor type 1 diabetes (T1D) PBMCs. *FLNA* virtual knockout predicted cell-context-dependent perturbation of immune-related transcriptional programs, including *IL4R* in DR B cells and *CTSD* in DR monocytes/NK cells. This prior-guided study identifies *FLNA* within a ten-gene GMMRG set as a circulating host-response signal that links curated microbe/metabolite–host records to immune-vascular and cytoskeletal remodeling in DR.

## 1. Introduction

Diabetic retinopathy (DR) is a leading microvascular complication of diabetes and a major cause of preventable vision loss [1,2]. Although retinal neurovascular injury is the clinical focus, DR also involves chronic metabolic stress, leukocyte–endothelial interactions, endothelial dysfunction and breakdown of the blood–retinal barrier [3,4,5,6,7,8,9,10,11,12,13,14]. Peripheral blood transcriptomics can capture systemic molecular changes associated with retinopathy among patients with diabetes.

The gut–retina axis has received increasing attention because gut microbes and microbial metabolites influence host immune tone, endothelial function and metabolic inflammation. Microbiota-derived metabolites, including short-chain fatty acids and bile acids, can circulate beyond the intestine and regulate immune and barrier-related host responses [15,16]. In DR, experimental studies have linked microbiome remodeling and bile-acid signaling to reduced retinal injury, while clinical and evidence-synthesis studies have reported gut microbial differences, longitudinal risk signals, Mendelian-randomization associations and altered circulating short-chain fatty acid profiles [17,18,19,20,21,22,23,24,25,26]. However, most DR transcriptomic datasets lack matched microbiome or metabolomic measurements.

gutMGene v2.0 curates experimentally and literature-supported associations among gut microbes, microbial metabolites and host genes [27,28]. We used gutMGene as a host-gene prior to annotate peripheral blood expression changes with reported microbe–host and metabolite–host relationships.

Cytoskeletal remodeling, cell adhesion and vascular-barrier regulation provide a plausible biological interface between systemic immune-metabolic stress and retinal microvascular injury [3,4,8,9,10,11,12,13,14,29,30,31,32]. Genes governing these processes may therefore capture transcriptional footprints of leukocyte–endothelial interaction, platelet or immune-cell activation, endothelial dysfunction and barrier disturbance in DR. Among cytoskeleton/adhesion-related genes, *FLNA*—which encodes the actin-binding scaffold protein filamin A—is of particular interest [33,34,35,36]. Filamin A organizes actin networks, coordinates cell adhesion and migration, transduces mechanical signals, and contributes to vascular remodeling [29,30,31,32,33,34,35,36]. These functions make *FLNA* a biologically plausible candidate whose expression in peripheral blood may reflect DR-associated vascular and cytoskeletal remodeling.

In this study, GSE221521 was used as the discovery cohort, and external blood/PBMC and retinal datasets were used to assess the tissue and cellular contexts of the resulting candidate signal [37]. The primary discovery comparison was restricted to DR versus diabetes without retinopathy (DM non-DR) to focus on retinopathy-associated variation within diabetes. We aimed to identify microbe/metabolite-annotated host genes associated with DR, refine them by co-expression module evidence and examine their relationship with immune-vascular and cytoskeletal programs. Because matched microbiome or metabolome profiles were unavailable in these transcriptomic cohorts, gutMGene was used as an annotation prior for host-gene interpretation.

## 2. Results

**Figure 1:**
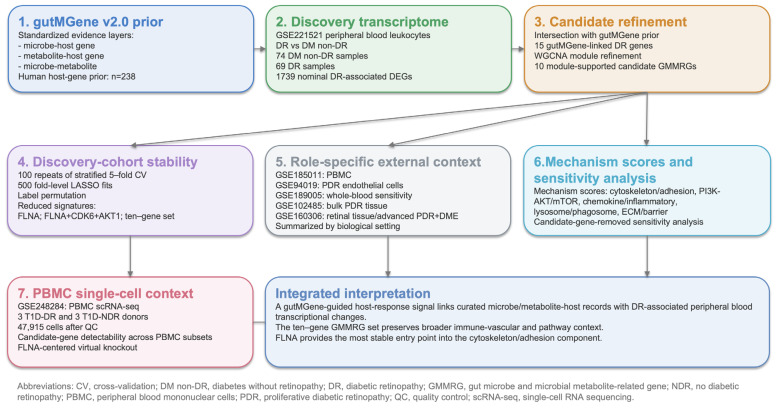
Analytical workflow and evidence hierarchy.

The overall analytical workflow and public datasets are summarized in the workflow and dataset table (Figure 1; Table 1).

### 2.1. gutMGene-Guided Screening Defined an Initial DR-Associated GMMRG Pool

**Figure 2:**
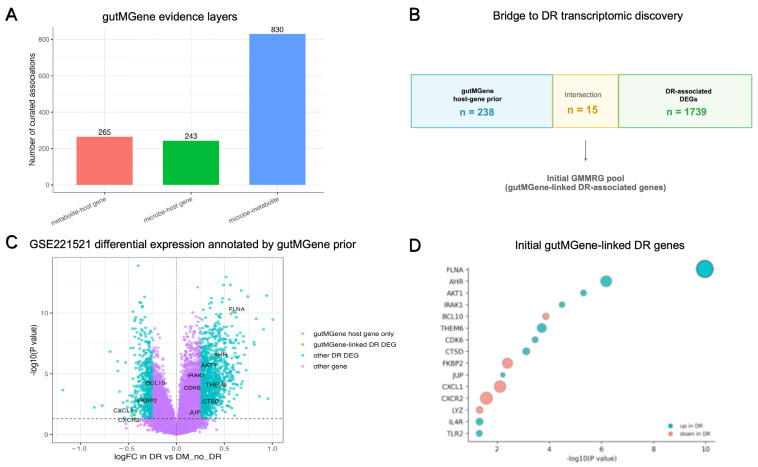
gutMGene host-gene prior and GSE221521 transcriptomic screening. (**A**) Number of curated microbe–host gene, metabolite–host gene and microbe–metabolite associations retained after standardization. (**B**) Intersection between the gutMGene-derived host-gene prior and DR-associated DEGs, yielding 15 initial GMMRGs. (**C**) DR versus diabetes without retinopathy volcano plot annotated by gutMGene-linked DR-associated genes. (**D**) Dot plot of the 15 initial gutMGene-linked DR genes.

The standardized gutMGene evidence set contained 243 microbe–host gene associations, 265 metabolite–host gene associations and 830 microbe–metabolite associations (Figure 2A). After deduplication, the gutMGene-derived host-gene prior contained 238 human genes. In GSE221521, 1739 genes met the nominal DR-associated DEG threshold in the DR versus DM non-DR comparison. The intersection between these two layers contained 15 gutMGene-linked DR-associated genes: *FLNA*, *AHR*, *AKT1*, *IRAK1*, *BCL10*, *THEM6*, *CDK6*, *CTSD*, *FKBP2*, *JUP*, *CXCL1*, *CXCR2*, *LYZ*, *IL4R* and *TLR2* (Figure 2B–D). In the volcano plot and initial-gene dot plot, *FLNA* showed the strongest upregulated signal, while *CXCL1* and *CXCR2* were among the downregulated gutMGene-linked DR-associated genes (Figure 2C,D).

### 2.2. WGCNA Refinement Retained Ten Final Candidate GMMRGs

**Figure 3:**
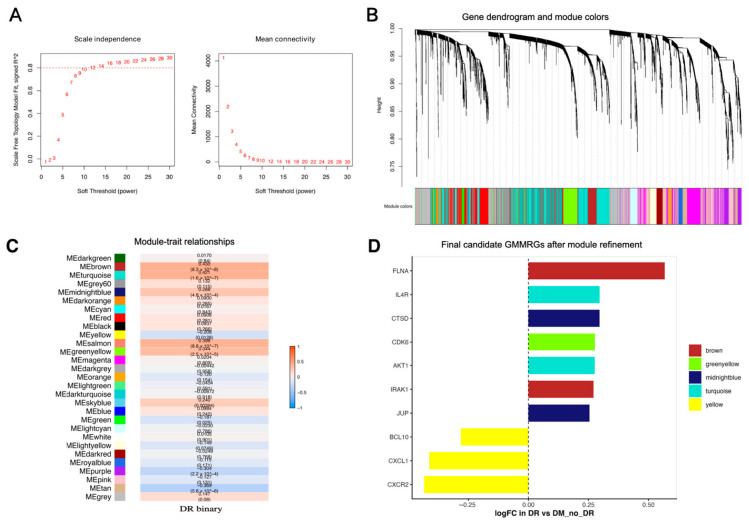
WGCNA module refinement and expression direction of final candidate GMMRGs. (**A**) Soft-threshold power selection showing scale-free topology fit and mean connectivity. (**B**) Hierarchical clustering dendrogram and module color assignment for the filtered expression matrix. The color strip below the dendrogram indicates WGCNA module assignment from dynamic tree cutting; each color denotes one co-expression module, and grey denotes genes not assigned to a non-grey module. (**C**) Module-trait correlation heatmap for DR status and related clinical grouping variables; values indicate module eigengene correlation coefficients with corresponding *p* values. (**D**) Module-colored logFC bar plot of the ten final candidate GMMRGs in DR versus diabetes without retinopathy.

WGCNA was performed in the 143 diabetes samples and identified 29 co-expression modules. Nine modules were associated with DR status at *p* < 0.05 and |correlation| ≥ 0.20, with the strongest positive correlations in brown (r = 0.430, *p* = 8.27 × 10^−8^), turquoise (r = 0.421, *p* = 1.60 × 10^−7^), salmon (r = 0.398, *p* = 8.77 × 10^−7^) and green-yellow (r = 0.344, *p* = 2.54 × 10^−5^) modules and negative correlations in tan, purple and yellow modules (Figure 3A–C). Module refinement reduced the 15 initial genes to ten module-supported candidate GMMRGs: *FLNA*, *AKT1*, *IRAK1*, *BCL10*, *CDK6*, *CTSD*, *JUP*, *CXCL1*, *CXCR2* and *IL4R* (Figure 3D; Table 2).

The ten candidates were distributed across five DR-associated modules: *FLNA* and *IRAK1* in brown; *AKT1* and *IL4R* in turquoise; *CDK6* in green-yellow; *CTSD* and *JUP* in midnight blue; and *BCL10*, *CXCL1* and *CXCR2* in yellow (Figure 3D). Each retained gene was gutMGene-linked, differentially expressed in DR and located in a DR-associated WGCNA module. The integrated evidence summary linked each candidate gene to its gutMGene evidence layer, DR-related expression change, module assignment and model-level role (Table 2; Figure S1). *FLNA* carried metabolite–host evidence and was upregulated in DR. *CXCR2* and *IL4R* carried microbe–metabolite–host triplet evidence, while the remaining candidates connected the set to chemokine, PI3K-AKT/mTOR, lysosomal and adhesion biology.

### 2.3. Discovery-Cohort Resampling Prioritized FLNA Within the Module-Supported Candidate Set

**Figure 4:**
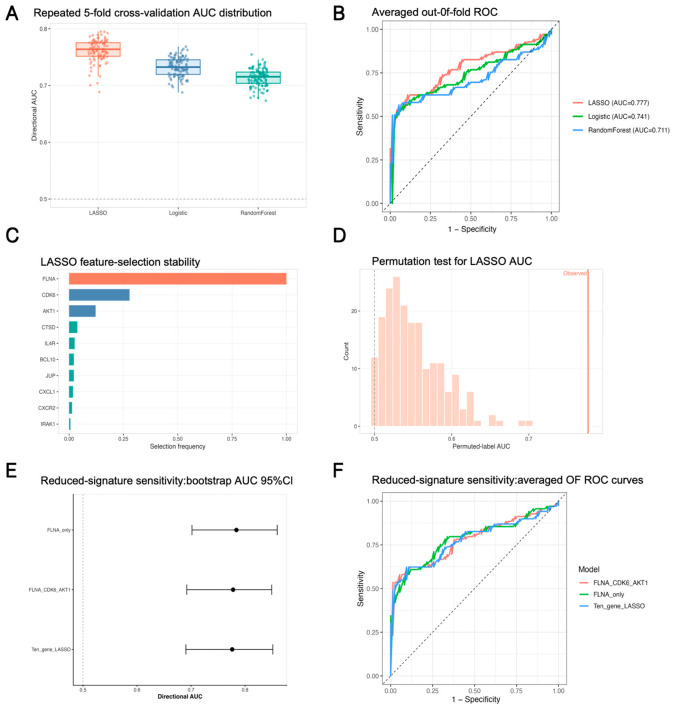
Discovery-cohort resampling and *FLNA*-associated reduced-signature analysis. (**A**) AUC distribution from 100 repeats of stratified five-fold cross-validation for logistic regression, LASSO and random forest. (**B**) Averaged out-of-fold ROC curves for the three models. (**C**) LASSO feature-selection stability across 500 fold-specific fits. (**D**) Permutation distribution of LASSO AUC with the observed AUC indicated by the vertical line. (**E**) Bootstrap AUC 95% confidence intervals for *FLNA*-only, *FLNA* + *CDK6* + *AKT1* and full ten-gene LASSO models. (**F**) Averaged out-of-fold ROC curves for reduced-signature analysis.

The ten candidate genes were evaluated by repeated resampling in the discovery cohort to assess robustness of the candidate signal. Logistic regression, LASSO and random forest were compared using out-of-fold probabilities, averaged AUCs and label permutation. LASSO achieved the highest averaged out-of-fold AUC (0.777; 95% CI, 0.696–0.851), followed by logistic regression (AUC 0.741) and random forest (AUC 0.711), and label permutation supported a non-random LASSO signal (permutation *p* = 0.00498; Figure 4A,B,D; Table 3).

LASSO selected *FLNA* in all 500 fold-specific fits (Figure 4C). *CDK6* and *AKT1* showed secondary selection frequencies (0.278 and 0.122), while the remaining candidates were rarely selected. In reduced-signature analysis, *FLNA* alone achieved an AUC of 0.784 (95% CI, 0.702–0.859), compared with 0.778 for *FLNA* + *CDK6* + *AKT1* and 0.776 for the full ten-gene LASSO model (Figure 4E,F; Table 4). These analyses prioritized *FLNA* as the most stable model-level candidate and the main cytoskeleton/adhesion-related entry point, while the full ten-gene GMMRG set was retained as the broader gutMGene-annotated host-response context.

### 2.4. External Blood/PBMC and Retinal Datasets Supported a Context-Dependent Candidate Signal

**Figure 5:**
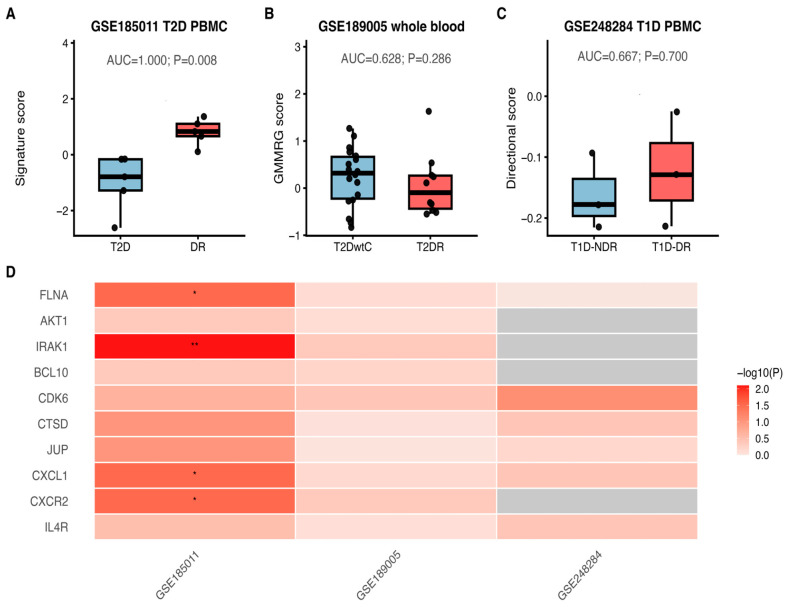
Candidate GMMRG score in external diabetic blood/PBMC datasets. (**A**) GMMRG score distribution in GSE185011 T2D PBMC samples comparing T2D without DR and DR samples. (**B**) GMMRG score distribution in GSE189005 T2D whole-blood samples comparing T2D without complications/T2DwtC and T2DR. (**C**) Donor-level GMMRG score distribution in GSE248284 T1D PBMC samples comparing T1D-NDR and T1D-DR donors. (**D**) Gene-level heatmap for the same external blood/PBMC datasets shown in (**A**–**C**). Red intensity indicates −log10(*p*); gray cells indicate unavailable, undetected or non-estimable gene-level statistics. Asterisks indicate nominal gene-level significance (* *p* < 0.05, ** *p* < 0.01).

External blood and PBMC datasets were examined using diabetic comparators where available. In GSE185011, the GMMRG score distinguished DR from T2D PBMC samples with complete separation (AUC = 1.000; *p* = 0.008; Figure 5A), supporting a strong PBMC signal in this T2D dataset despite the small cohort size. In GSE189005 whole blood, separation between T2DR and T2D without complications/T2DwtC was weaker (AUC = 0.628; *p* = 0.286; Figure 5B). In GSE248284, donor-level summaries in the six-donor T1D PBMC dataset showed limited DR-NDR separation (AUC = 0.667; *p* = 0.700; Figure 5C). Gene-level heatmap analysis showed the densest candidate-level signal in GSE185011, with fewer nominal gene-level changes in GSE189005 and GSE248284 (Figure 5D). Dataset-level score statistics and detectable-candidate counts are provided in Supplementary Table S1.

Retinal endothelial-cell and retinal tissue datasets then extended the assessment across tissue contexts. In GSE94019, the GMMRG score separated PDR from control retinal endothelial cells with complete separation (AUC = 1.000; *p* = 0.003; Figure S2A), supporting an endothelial-enriched context for the candidate signal despite the small sample size. In GSE102485, T2D-PDR neovascular membrane samples and the smaller T1D-PDR subset separated from normal retinal controls (T2D-PDR: AUC = 0.789, *p* = 0.132; T1D-PDR: AUC = 0.778, *p* = 0.400; Figure S2B,C). In GSE160306, a diabetes-subtype-unresolved human retinal tissue dataset, broad DR-stage samples showed limited separation from diabetic retinal tissue without retinopathy, whereas the advanced PDR with diabetic macular edema (PDR + DME) subset showed clearer score elevation (Figure S2D,E). Retinal and endothelial gene-level heatmaps showed which candidate genes were detectable and estimable in each tissue context, and Supplementary Table S1 lists the corresponding dataset-level score statistics and detectable-candidate counts (Figure S2F).

Overall, the candidate signal was strongest in T2D PBMCs, retinal endothelial cells and advanced PDR + DME retinal tissue, whereas whole blood, broad retinal tissue and the six-donor T1D PBMC analysis showed more limited separation. These results support tissue-, cell-type- and disease-stage dependence of the candidate signal.

### 2.5. Mechanism Scores Annotated the Candidate Set with Vascular/Barrier and Immune-Chemotactic Biology

**Figure 6:**
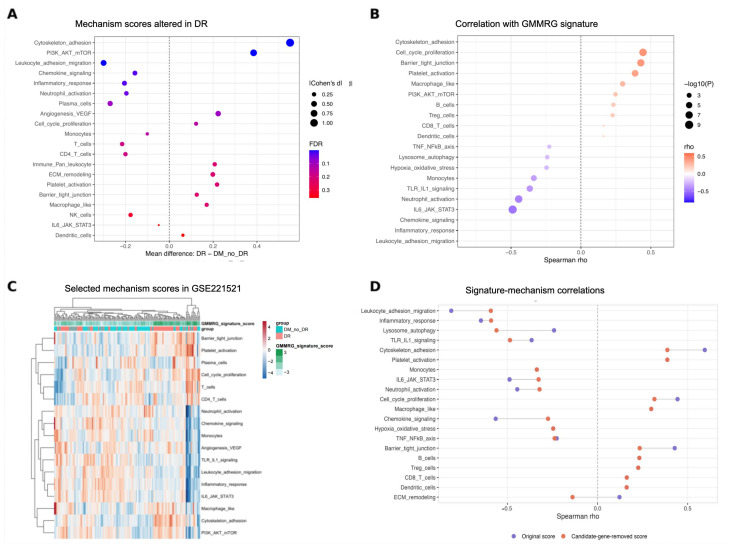
Mechanism-oriented gene-set scoring and candidate-gene-removed sensitivity analysis. (**A**) Mechanism score differences between DR and DM non-DR samples, with effect size and FDR summarized for each score. (**B**) Spearman correlations between the GMMRG score and mechanism scores. (**C**) Correlation heatmap showing the relationship among the GMMRG score and major mechanism scores. (**D**) Candidate-gene-removed sensitivity analysis showing the correlation structure after direct candidate-gene overlap was removed from scored gene sets.

Two mechanism-score directions were observed in the discovery cohort. Cytoskeleton-adhesion and PI3K-AKT/mTOR scores were increased in DR (Cohen d = 1.10 and 0.72; FDR = 8.31 × 10^−8^ and 4.65 × 10^−5^), whereas leukocyte adhesion/migration, inflammatory response, chemokine signaling, phagosome and extracellular matrix (ECM)–receptor interaction scores were lower in DR (absolute Cohen d = 0.56–1.02; FDR ≤ 1.64 × 10^−3^; Figure 6A).

The GMMRG signature score showed strong inverse correlations with leukocyte adhesion/migration (rho = −0.812), inflammatory response (rho = −0.647) and chemokine signaling (rho = −0.565), and positive correlations with cytoskeleton-adhesion (rho = 0.595), cell-cycle/proliferation (rho = 0.444) and barrier/tight-junction scores (rho = 0.428) (Figure 6B,C). The main correlation structure persisted after removing the ten candidate genes from the scoring sets (Figure 6D), indicating that the pathway-level signal was not driven only by direct inclusion of the candidate genes in the same scores.

### 2.6. PBMC Single-Cell Localization and FLNA-Centered Virtual Knockout Analysis

PBMC scRNA-seq analysis was used to localize candidate-gene expression across circulating immune-cell subsets. After quality control, 47,915 cells from six T1D donors were retained and resolved into putative PBMC cell types, including T cells, NK cells, monocytes, B cells, platelets, plasma cells and dendritic cells (Figure S3A,B). Candidate GMMRG expression was detectable in selected PBMC subsets; *FLNA* and CTSD showed relatively broad detectability, *CDK6* was most evident in dendritic-cell-like cells, and *CXCL1* and *CXCR2* were sparse in the retained PBMC object (Figure S3C). *FLNA* was detectable across multiple PBMC subsets, with higher detectability in monocytes and NK cells, providing the cellular context for *FLNA*-centered virtual knockout analysis (Figure S3D).

*FLNA*-centered virtual knockout was performed using NDR PBMC reference networks to simulate *FLNA* loss in single-cell GRNs, and DR networks were analyzed in parallel for disease-state comparison. Mixed AllPBMC networks yielded few FDR-significant non-*FLNA* perturbed genes, whereas cell-type-specific networks showed stronger perturbation burdens, especially in monocytes and NK cells (Figure S4A).

*FLNA* virtual knockout affected selected candidate GMMRGs in a cell-context-specific manner. *IL4R* was significantly affected in DR B cells, while *CTSD* was significantly affected in DR monocytes and DR NK cells (Figure S4B,C). Curated module-overlap analysis suggested that the predicted perturbation profiles were enriched for cytotoxic/NK, myeloid inflammatory, antigen-presentation and lysosome/phagosome-related modules (Figure S4D).

## 3. Discussion

*FLNA* emerged as the most robustly prioritized candidate within the gutMGene-linked DR host-gene set, anchoring a cytoskeleton/adhesion-centered signal that connected peripheral blood transcriptomics with immune-vascular and vascular-barrier biology. The ten-gene set retained broader gutMGene evidence through metabolite–host and microbe–metabolite–host annotations, while *FLNA* provided the most stable entry point into the cytoskeleton/adhesion component of this signal.

The gut–retina axis provides the biological rationale for using gutMGene as a host-gene prior. Gut microbes and microbial metabolites can regulate systemic immune responses, endothelial function and barrier-related host biology [38,39,40,41,42,43,44]. In DR, experimental and clinical studies have reported microbiome remodeling, bile-acid signaling changes, longitudinal microbial risk signatures and altered circulating short-chain fatty acid profiles [17,18,19,20,21,22,23,24,25,26]. In the present analysis, gutMGene annotated DR-associated host transcriptional changes with curated microbe/metabolite–host records [27,28], while microbial abundance and metabolite levels were not directly profiled in the analyzed patients.

The prioritized genes point to a coherent immune-vascular and cytoskeletal biology. *FLNA* is an actin-binding scaffold involved in cytoskeletal organization, adhesion, mechanotransduction, receptor signaling and vascular remodeling [29,30,31,32,33,34,35,36,45,46,47,48,49,50]. These functions are relevant to DR because endothelial permeability, leukocyte–endothelial interaction and vascular remodeling depend on coordinated cytoskeletal and adhesion responses [3,4,8,9,10,11,12,13,14,29,30,31,32]. The remaining candidates extend this signal toward chemokine signaling, PI3K-AKT/mTOR activity, lysosomal biology and immune regulation [45,46,47,48,49,50]. *FLNA* should therefore be interpreted as a prioritized host-response candidate within a cytoskeleton/adhesion-centered axis, not as a direct microbial biomarker.

The external dataset pattern was also consistent with a context-dependent host-response signal. The strongest separation appeared in T2D PBMCs and retinal endothelial cells, which match the circulating immune and vascular components emphasized by the candidate GMMRG set. Weaker separation in GSE189005 whole blood may reflect lower cellular resolution and dilution of PBMC-enriched immune-vascular signals within a mixed blood-cell transcriptome. In retinal tissue datasets, the signal was not uniformly distributed across all comparisons; it was clearer in endothelial-cell and advanced PDR + DME contexts than in broader retinal-stage comparisons. Bulk PDR neovascular membranes and retinal tissue contain mixed vascular, stromal, glial and inflammatory components, which can attenuate a signal that is more evident in PBMC, endothelial-enriched or advanced retinal disease contexts. This pattern supports a tissue-, cell-type- and disease-stage-dependent interpretation rather than a single uniform classifier across all blood and retinal sources.

Mechanism-score analysis helped clarify how the candidate set behaved in bulk peripheral blood. Cytoskeleton-adhesion and PI3K-AKT/mTOR scores were higher in DR, consistent with the *FLNA*-centered signal. By contrast, lower leukocyte-adhesion and inflammatory scores likely reflect differences in circulating immune-cell composition or activation states, including monocyte or T-cell subset shifts that bulk blood transcriptomes cannot fully resolve [11,12,51].

These findings motivated a single-cell resolution analysis to resolve the cellular origins of the signal. The candidate genes were not uniformly distributed across PBMC subsets. *FLNA* and *CTSD* showed broad detectability, whereas *CXCL1* and *CXCR2* were sparse in the retained PBMC object, indicating that the ten-gene signal is carried by distinct immune-cell backgrounds rather than by a homogeneous blood-wide expression program. The higher *FLNA* detectability in monocytes and NK cells was particularly relevant because these cell types participate in inflammatory surveillance, endothelial interaction and tissue-trafficking programs that are closely linked to diabetic microvascular injury [11,12,51]. Thus, the single-cell analysis positioned *FLNA* within specific circulating immune-cell contexts in which its regulatory effects could be interrogated.

The scTenifoldKnk analysis extended this localization result from expression detectability to predicted regulatory consequence. Mixed PBMC networks showed limited perturbation after *FLNA* virtual knockout, whereas cell-type-specific networks showed broader predicted effects, especially in monocytes and NK cells. This contrast suggests that the *FLNA*-associated signal is diluted when PBMCs are modeled as a mixed population but becomes more apparent within specific immune-cell regulatory networks. At the candidate-gene level, the predicted perturbation was selective: *IL4R* was affected in DR B cells, while *CTSD* was affected in DR monocytes and NK cells. This pattern links *FLNA*-centered cytoskeletal remodeling to immune-receptor signaling in B-cell contexts and lysosomal/phagosome-related programs in monocyte and NK-cell contexts. The module-overlap results further support this interpretation, with enrichment in cytotoxic/NK, myeloid inflammatory, antigen-presentation and lysosome/phagosome-related modules.

Together, the PBMC single-cell and virtual-knockout analyses indicate that *FLNA* is not simply a bulk blood marker. Instead, *FLNA* marks a cell-context-dependent regulatory axis that connects cytoskeletal organization with immune-cell programs relevant to DR-associated vascular injury. These predicted *FLNA–IL4R* and *FLNA–CTSD* relationships provide experimentally testable links for future PBMC-based and retinal vascular functional studies. Importantly, the GSE248284 analysis was not intended to infer T1D-specific disease mechanisms, but rather to assess whether the prioritized cytoskeleton/adhesion-centered signal could be localized to, and exert regulatory effects within, defined PBMC subsets, independent of the upstream diabetic etiology. Although T1D and T2D may differ in upstream autoimmune and metabolic drivers, DR in both settings involves downstream immune-vascular injury, including circulating immune-cell activation and leukocyte–endothelial interaction. In this sense, GSE248284 provided cellular-context evidence for the candidate signal, while larger T1D- and T2D-stratified PBMC scRNA-seq cohorts will be required to determine whether the *FLNA*-centered regulatory patterns are shared across diabetes subtypes or subtype-specific.

Several limitations should be noted. First, GSE221521 served as both the discovery cohort and the source for internal robustness analyses, including WGCNA refinement, resampling, LASSO stability testing, and reduced-signature evaluation. Importantly, diabetes subtype was not annotated at the sample level in this cohort. Consequently, the present analysis identifies a DR-associated host-response signal across a mixed diabetic population, but cannot determine whether the same upstream immune-metabolic drivers operate equivalently in T1D and T2D. Second, the external validation datasets were heterogeneous in sample source, sample size, and phenotype definitions, which complicates direct cross-dataset comparisons. Third, matched microbiome and metabolomic profiles were unavailable; therefore, the gutMGene layer served as a literature-derived annotation prior rather than as a direct measurement of microbial or metabolic exposure. Finally, as a computational and associative study, this analysis cannot establish causal or temporal relationships among gut-microbial signals, host-gene expression and DR progression. Nevertheless, the prioritized candidates, particularly *FLNA*, provide a focused set for functional interrogation. Future validation in retinal endothelial cells, immune-cell models and in vivo DR animal models will be required to determine whether *FLNA* and the associated candidate genes contribute mechanistically to diabetic retinal injury. Future paired multi-omics cohorts, incorporating diabetes subtype, detailed retinal phenotyping, microbiome, metabolomics and host transcriptomics from the same individuals, will further complement these functional efforts to rigorously test the *FLNA*-centered candidate set in T1D- and T2D-stratified populations.

## 4. Materials and Methods

### 4.1. Study Design and Datasets

This study used public datasets and curated database resources; no new human samples were collected. GSE221521 was used as the discovery cohort for integrating gutMGene-derived microbe/metabolite–host gene evidence with peripheral blood transcriptomics [52]. Public external blood/PBMC and retinal datasets were analyzed according to sample source and disease group.

All computational analyses were implemented with scripted R and Python workflows. The final single-cell virtual-knockout analysis used R 4.5.2, Seurat 5.4.0 and scTenifoldKnk 1.0.3; Python figure generation used Python 3.12 with pandas 2.3.3, NumPy 2.4.1 and matplotlib 3.10.8. Package versions and sessionInfo outputs for the differential-expression, WGCNA, modeling and single-cell pipelines are provided in the Supplementary Code/analysis archive.

### 4.2. gutMGene Prior and Differential-Expression Screening

The gutMGene files were standardized into microbe–host gene, metabolite–host gene and microbe–metabolite association tables. Human host genes appearing in microbe–host or metabolite–host records were combined and deduplicated to generate the host-gene prior [27,28]. These curated associations defined the annotation layer used to prioritize host transcriptomic signals in GSE221521. Differential expression in GSE221521 used normalized FPKM values. Columns ending with `FPKM` were retained for the primary analysis. Gene symbols were extracted from the gene-name annotation column, and duplicate gene symbols were collapsed by averaging expression values. The FPKM matrix was transformed to log2(FPKM + 1) when the expression-value distribution indicated non-log-transformed normalized values. Genes were filtered before modeling by retaining genes expressed above log2(0.1 + 1) in at least 20% of samples and with nonzero variance. Differential expression was performed with limma::lmFit followed by empirical Bayes moderation using limma::eBayes [53]. The primary contrast was DR versus DM non-DR. Genes with nominal *p* < 0.05 and |logFC| ≥ 0.25 were used for candidate screening. Adjusted *p* values were retained in the output tables. The initial GMMRG pool was defined as the intersection between DR-associated DEGs and the gutMGene host-gene prior.

### 4.3. WGCNA Module Refinement and Candidate Prioritization

WGCNA was performed in the 143 diabetes samples used for the primary comparison [54]. These 143 samples were obtained from the GSE221521 dataset and consisted of the 74 diabetes-without-retinopathy and 69 DR samples used in the primary comparison. The expression input contained 8098 genes after filtering and quality control. A signed bicor network was constructed. The soft-thresholding power was selected according to scale-free topology and mean connectivity diagnostics. Candidate GMMRGs were retained when they were gutMGene-linked, differentially expressed in DR and located in modules associated with DR status.

WGCNA used a signed bicor network (networkType = ‘signed’, corType = ‘bicor’), soft-thresholding power of 10 selected from scale-free topology and mean connectivity diagnostics, minimum module size of 30 and module merging at mergeCutHeight = 0.25. Module–trait associations were evaluated against DR status, and modules with *p* < 0.05 and |correlation| ≥ 0.20 were considered DR-associated for candidate refinement. WGCNA modules were interpreted as co-expression groups and were not assumed to indicate structural similarity or identical function among member genes.

### 4.4. Discovery-Cohort Resampling, External Dataset Analysis and Mechanism Scoring

Discovery-cohort signal stability was assessed with 100 repeats of stratified five-fold cross-validation. Preprocessing, model fitting and prediction were performed inside the resampling procedure. Logistic regression, LASSO and random forest were compared using averaged out-of-fold probabilities, bootstrap AUC estimates and repeated cross-validation summaries [55,56,57]. Label permutation testing was performed to assess whether the observed LASSO signal exceeded the random-label expectation. Reduced-signature sensitivity analysis compared *FLNA*-only, *FLNA + CDK6 + AKT1* and ten-gene LASSO models. The ten-gene set included *FLNA*, *AKT1*, *IRAK1*, *BCL10*, *CDK6*, *CTSD*, *JUP*, *CXCL1*, *CXCR2* and *IL4R*. For GSE189005, samples annotated as T2D without complications/T2DwtC were used as diabetic non-DR comparators, and T2DR samples were used as DR cases. Healthy controls were excluded because the analysis was designed as a diabetes-internal DR versus non-DR comparison, and T2DN samples were excluded to avoid diabetic nephropathy-related confounding. The exact GSM IDs, original GEO annotations, assigned groups, inclusion status and reasons for inclusion or exclusion are provided in Supplementary Table S2.

LASSO used alpha = 1, with lambda selected within training folds during cross-validation. Random forest used 500 trees, and AUCs with 95% confidence intervals were calculated with pROC. Candidate GMMRG scores were calculated as the mean of z-standardized expression values for detectable candidate genes within each dataset. When candidate genes were unavailable in an external dataset, the score used the detectable subset and the number of detectable genes was reported in Supplementary Table S1. Between-group comparisons used Wilcoxon rank-sum tests unless otherwise stated, and multiple testing used Benjamini–Hochberg FDR correction.

### 4.5. PBMC Single-Cell Processing and scTenifoldKnk Virtual-Knockout Analysis

GSE248284 PBMC scRNA-seq data were used for single-cell analysis [51,58]. Six T1D donors were included, consisting of three T1D-DR and three T1D without DR (T1D-NDR) donors. Standard quality control, normalization, dimensionality reduction, clustering and marker-based annotation were performed to define putative PBMC cell types.

*FLNA*-centered single-cell virtual knockout analysis was performed using scTenifoldKnk [59]. NDR cells were used as the reference input for the main PBMC GRN analysis, and DR cells were analyzed in parallel as a disease-state comparison. Global PBMC and cell-type-specific networks were constructed when sufficient cells and detectable *FLNA* expression were available; cell-type-specific runs required at least 300 cells per disease-state stratum. For each run, up to 1500 cells and 3000 genes were retained after detectability filtering, while *FLNA* and candidate GMMRGs were retained when present. scTenifoldKnk constructed 10 single-cell GRNs per run, using up to 500 cells per network, nComp = 3 and tensor decomposition K = 3. *FLNA* knockout was simulated by removing *FLNA* outgoing regulatory edges from the reconstructed network. Differentially regulated genes after virtual knockout were ranked using the manifold-alignment statistics provided by scTenifoldKnk, with Benjamini–Hochberg FDR adjustment. The resulting ranked perturbation profiles were used as computational GRN predictions for downstream interpretation.

For DR-NDR summaries in GSE248284, module scores were aggregated at the donor level and at the donor-by-cell-type level before statistical testing to reduce cell-level pseudo-replication. Cell-type strata with very small cell counts were summarized descriptively. The six-donor design limited statistical power for disease-state inference.

GSE160306 was processed as a diabetes-subtype-unresolved human retinal tissue total RNA-seq dataset. The processed normalized CPM matrix was log2-transformed, and explicit GEO characteristics were used to define healthy control, diabetic without retinopathy, NPDR, NPDR + DME and PDR + DME retinal tissue groups. Because T1D/T2D labels were not consistently available, GSE160306 was not used for diabetes-subtype-specific validation. Candidate GMMRG score and gene-level summaries were calculated from the eight detectable candidate genes in the processed matrix (*FLNA*, *AKT1*, *IRAK1*, *BCL10*, *CDK6*, *CTSD*, *JUP* and *IL4R*); *CXCL1* and *CXCR2* were not detected.

## 5. Conclusions

This study identifies *FLNA* as a prioritized candidate within a ten-gene gutMGene-linked host-response set in DR. The candidate signal connects curated microbe/metabolite–host records with peripheral immune-vascular and cytoskeleton/adhesion biology, and it is most evident in T2D PBMCs, retinal endothelial cells and advanced PDR + DME retinal tissue. Single-cell GRN perturbation further localized *FLNA*-centered effects to selected immune-cell contexts, including predicted *IL4R* perturbation in DR B cells and *CTSD* perturbation in DR monocytes and NK cells. These findings provide a focused host-gene set for paired microbiome, metabolomic, transcriptomic and functional studies of DR.

## Figures and Tables

**Table 1:** Public datasets included in this study.

Dataset	Source	Groups Used	Role
GSE221521 https://www.ncbi.nlm.nih.gov/geo/query/acc.cgi?acc=GSE221521 (accessed on 15 May 2026)	Peripheral blood leukocytes	74 diabetes without retinopathy and 69 DR; 50 healthy controls were not used in the primary comparison	Discovery cohort for differential expression, WGCNA, model construction and internal robustness analyses
GSE185011 https://www.ncbi.nlm.nih.gov/geo/query/acc.cgi?acc=GSE185011 (accessed on 15 May 2026)	PBMC	5 T2D and 5 T2D-DR samples	External T2D PBMC score and gene-level assessment
GSE189005 https://www.ncbi.nlm.nih.gov/geo/query/acc.cgi?acc=GSE189005 (accessed on 15 May 2026)	Whole blood cells	18 T2D without complications/T2DwtC and 10 T2D-DR samples	External T2D whole-blood score assessment
GSE248284 https://www.ncbi.nlm.nih.gov/geo/query/acc.cgi?acc=GSE248284 (accessed on 15 May 2026)	PBMC scRNA-seq	3 T1D-NDR and 3 T1D-DR donors; 47,915 cells retained after QC	T1D PBMC donor-level and single-cell analysis
GSE94019 https://www.ncbi.nlm.nih.gov/geo/query/acc.cgi?acc=GSE94019 (accessed on 15 May 2026)	Retinal endothelial cells	4 control endothelial and 9 PDR endothelial samples	Retinal endothelial-cell score assessment
GSE102485 https://www.ncbi.nlm.nih.gov/geo/query/acc.cgi?acc=GSE102485 (accessed on 15 May 2026)	Retinal tissue/PDR neovascular membrane	3 normal retinal controls, 19 T2D-PDR and 3 T1D-PDR samples	PDR tissue score and gene-level assessment
GSE160306 https://www.ncbi.nlm.nih.gov/geo/query/acc.cgi?acc=GSE160306 (accessed on 15 May 2026)	Human retinal tissue total RNA-seq	20 diabetic retinal tissues without retinopathy and 36 DR-stage retinal tissues, including 5 advanced PDR + DME tissues	Retinal tissue stage and advanced PDR + DME analysis

**Table 2:** Layered gutMGene, transcriptomic, model role and mechanistic evidence for the ten final candidate GMMRGs.

Gene	gutMGene Evidence Layer	logFC	WGCNA Module	Model Role	Mechanistic Annotation
*FLNA*	metabolite–host (urolithin A)	0.567	brown	selected in 500/500 LASSO fits	cytoskeleton/adhesion
*AKT1*	metabolite–host	0.275	turquoise	secondary model support	PI3K-AKT signaling
*IRAK1*	microbe–host (*Escherichia coli*)	0.271	brown	mechanistic support	TLR/IL-1 signaling
*BCL10*	microbe–host (*Lactobacillus acidophilus*)	−0.282	yellow	mechanistic support	NF-κB/immune signaling
*CDK6*	metabolite–host	0.277	green-yellow	secondary model support	cell-cycle signaling
*CTSD*	metabolite–host (urolithin A)	0.296	midnight blue	mechanistic support	lysosomal/extracellular-matrix biology
*JUP*	microbe–host (*Lactiplantibacillus plantarum*)	0.254	midnight blue	mechanistic support	adhesion/barrier
*CXCL1*	microbe–host (*Collinsella*)	−0.413	yellow	mechanistic support	chemokine signaling
*CXCR2*	metabolite–host; microbe–metabolite–host triplet	−0.434	yellow	mechanistic support	chemokine receptor/neutrophil axis
*IL4R*	metabolite–host; microbe–metabolite–host triplet	0.296	turquoise	mechanistic support	IL-4/IL-13 immune regulation

**Table 3:** Internal model performance in GSE221521.

Model	OOF AUC	95% CI Low	95% CI High	Repeated CV Mean	Repeated CV SD
LASSO	0.777	0.696	0.851	0.762	0.020
Logistic	0.741	0.652	0.821	0.733	0.017
Random forest	0.711	0.614	0.796	0.714	0.016

**Table 4:** Reduced-signature sensitivity analysis.

Reduced Model	AUC	95% CI Low	95% CI High	Permutation P
*FLNA*-only	0.784	0.702	0.859	0.005
*FLNA* + *CDK6* + *AKT1*	0.778	0.692	0.849	0.005
Ten-gene LASSO	0.776	0.690	0.851	0.005

Note: The ten-gene set included *FLNA*, *AKT1*, *IRAK1*, *BCL10*, *CDK6*, *CTSD*, *JUP*, *CXCL1*, *CXCR2* and *IL4R*.

## Data Availability

All datasets analyzed in this study are publicly available from the NCBI Gene Expression Omnibus (GEO): GSE221521, GSE185011, GSE189005, GSE248284, GSE94019, GSE102485 and GSE160306. The gutMGene v2.0 files are publicly available from the gutMGene database. All scripts used for data processing, figure generation and revision analyses are provided in the analysis archive.

## Supplementary Materials

The following supporting information can be downloaded at https://www.mdpi.com/article/10.3390/ijms27146182/s1.

## Abbreviations

The following abbreviations are used in this manuscript:

AUC	area under the receiver operating characteristic curve
CD31	cluster of differentiation 31
CI	confidence interval
CV	cross-validation
DEG	differentially expressed gene
DME	diabetic macular edema
DM non-DR	diabetes without retinopathy
DR	diabetic retinopathy
ECM	extracellular matrix
FDR	false discovery rate
FLNA	filamin A
FPKM	fragments per kilobase of transcript per million mapped reads
GEO	Gene Expression Omnibus
GMMRG	gut microbe and microbial metabolite-related gene
GO	Gene Ontology
GRN	gene regulatory network
KO	knockout
LASSO	least absolute shrinkage and selection operator
logFC	log fold change
NDR	no diabetic retinopathy
NK cell	natural killer cell
PBMC	peripheral blood mononuclear cell
PCA	principal component analysis
PDR	proliferative diabetic retinopathy
QC	quality control
RNA-seq	RNA sequencing
ROC	receiver operating characteristic
SCFA	short-chain fatty acid
scRNA-seq	single-cell RNA sequencing
T1D	type 1 diabetes
T2D	type 2 diabetes
UMAP	uniform manifold approximation and projection
WGCNA	weighted gene co-expression network analysis
