# Supplementary material for: GutMGene-Guided Peripheral Blood Transcriptomics Identifies an FLNA-Associated Host-Gene Signal in Diabetic Retinopathy
Source: Int J Mol Sci. 2026 Jul 10;27(14):6182. doi: 10.3390/ijms27146182 (PMC13409884; doi:10.3390/ijms27146182)
Supplement: Supplementary file 1 [file ijms-27-06182-s001.zip › ijms-4380386-supplementary.pdf]

Supplementary Materials

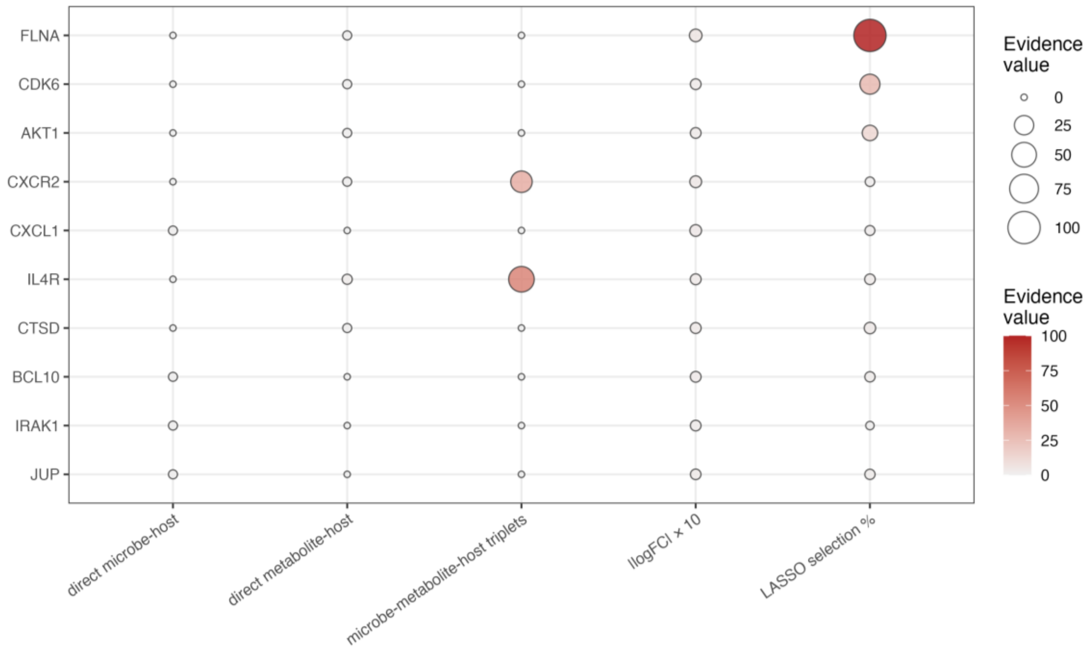

Figure S1. Layered evidence for the ten final candidate GMMRGs.

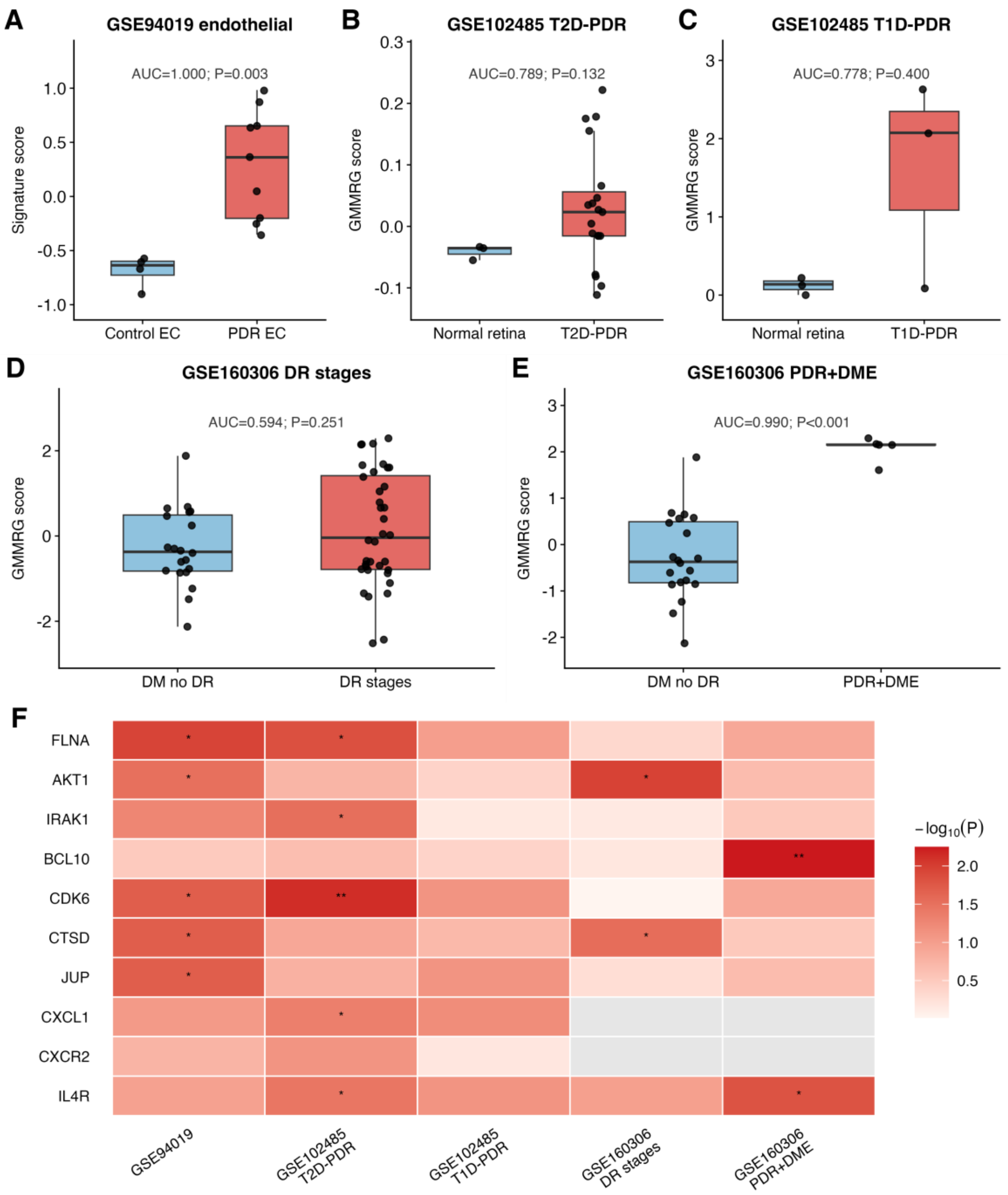

Figure S2. Retinal endothelial-cell and retinal tissue analyses. A, GMMRG score distribution in GSE94019 retinal endothelial cells comparing control endothelial and PDR endothelial samples. B, GMMRG score distribution in GSE102485 comparing normal retinal controls and T2D-PDR tissue samples. C, GMMRG score distribution in GSE102485 comparing normal retinal controls and T1D-PDR tissue samples. D, available-candidate GMMRG score distribution in GSE160306 comparing diabetic retinal tissue without retinopathy and broad DR-stage retinal tissue. E, available-candidate GMMRG score distribution in GSE160306 comparing diabetic retinal tissue without retinopathy and advanced PDR+DME retinal tissue. F, gene-level heatmap across the retinal endothelial and retinal tissue settings. For

GSE160306, analyses used the eight detectable candidate genes available in the processed matrix; CXCL1 and CXCR2 were not detected.

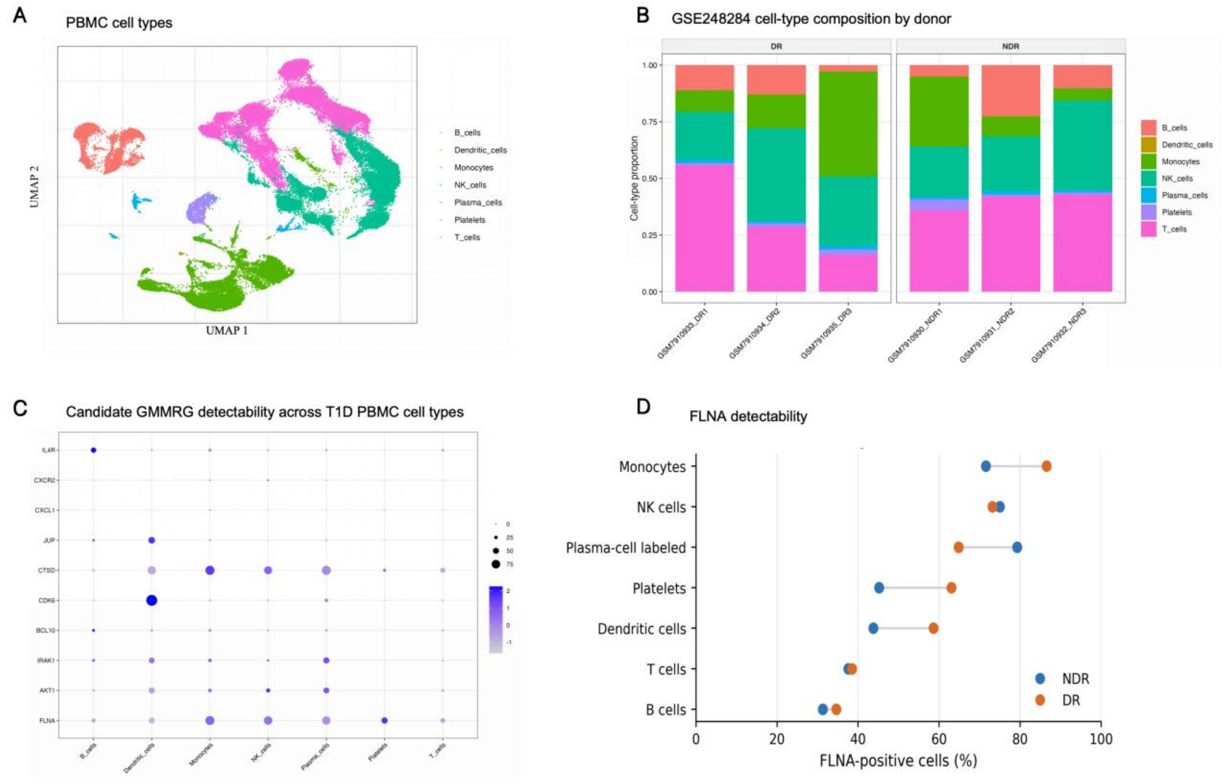

Figure S3. PBMC single-cell localization in GSE248284. A, UMAP of PBMC cell types. B, cell-type composition by donor and disease group. C, candidate GMMRG expression across PBMC cell types. D, FLNA detectability in NDR and DR donors. Dendritic cells are shown for localization but were not used for virtual-knockout runs because of low cell numbers.

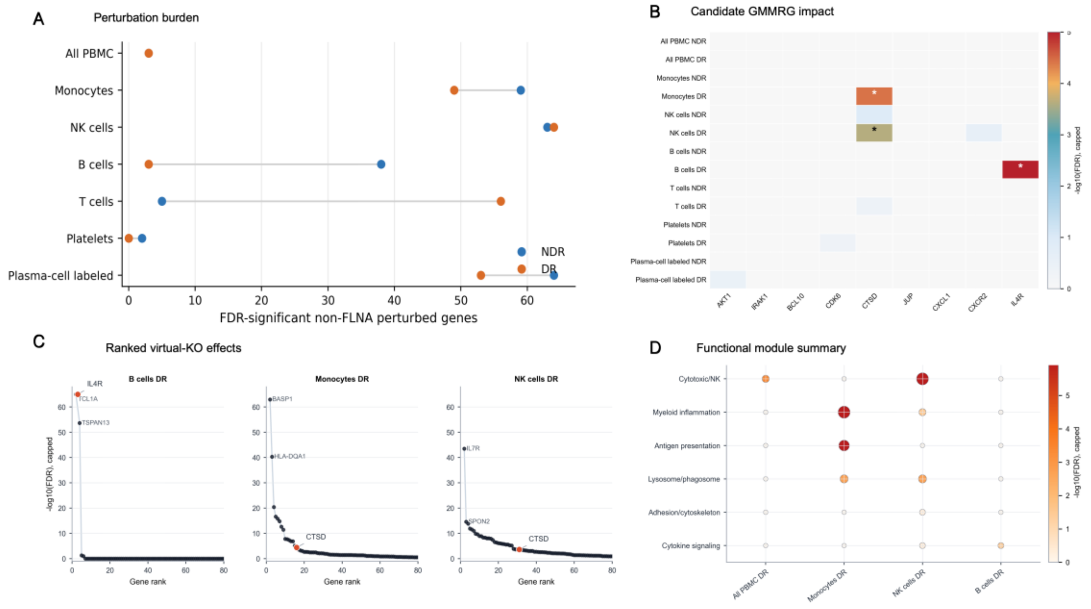

Figure S4. FLNA-centered scTenifoldKnk virtual knockout analysis in GSE248284 PBMC scRNA-seq data. A, number of FDR-significant non-FLNA genes predicted to be perturbed after FLNA virtual knockout across global PBMC and cell-type-specific networks. B, candidate GMMRG perturbation profile after FLNA virtual knockout. Color indicates capped -log10(FDR), and asterisks indicate FDR < 0.05. C, ranked virtual-knockout effects in key DR cell contexts, highlighting IL4R in DR B cells and CTSD in DR monocytes and DR NK cells. D, overlap between predicted perturbed genes and curated functional modules.

Supplementary Table S1. External score analyses across blood/PBMC, retinal endothelial-cell and retinal tissue datasets.

**Dataset**	**Sample source**	**Comparison**	**Comparator / control n**	**Case n**	**AUC**	**P value**	**Figure / result summary**
GSE185011	T2D PBMC	T2D vs DR	5	5	1.000	0.008	Figure 5A; T2D PBMC separation
GSE189005	T2D whole blood	T2D without complications/T2DwtC vs T2DR	18	10	0.628	0.286	Figure 5B; limited T2D whole-blood separation
GSE248284	T1D PBMC scRNA-seq	T1D-NDR vs T1D-DR	3 donors	3 donors	0.667	0.700	Figure 5C; limited six-donor T1D PBMC separation
GSE94019	Retinal endothelial cells	Control endothelial vs PDR endothelial	4	9	1.000	0.003	Figure S2A; endothelial-cell separation
GSE102485 T2D-PDR	PDR neovascular membrane / normal retina	Normal retina vs T2D-PDR tissue	3	19	0.789	0.132	Figure S2B; T2D-PDR tissue separation
GSE102485 T1D-PDR	PDR neovascular membrane / normal retina	Normal retina vs T1D-PDR tissue	3	3	0.778	0.400	Figure S2C; T1D-PDR tissue separation with small sample size
GSE160306 DR stages	Human retinal tissue	Diabetes without retinopathy vs DR-stage retinal tissue	20	36	0.594	0.251	Figure S2D; limited broad DR-stage separation
GSE160306 PDR+DME	Human retinal tissue	Diabetes without retinopathy vs advanced PDR+DME retinal tissue	20	5	0.990	<0.001	Figure S2E; advanced PDR+DME separation

Supplementary Table S2. Sample-level grouping for GSE189005.

**GSM ID**	**Original GEO annotation**	**Assigned group**	**Included**	**Reason**

GSM5693105	Control - Clustering group A - #1	Excluded	No	Healthy control; not a diabetic non-DR comparator
GSM5693106	Control - Clustering group A - #2	Excluded	No	Healthy control; not a diabetic non-DR comparator
GSM5693107	Control - Clustering group A - #3	Excluded	No	Healthy control; not a diabetic non-DR comparator
GSM5693108	Control - Clustering group A - #4	Excluded	No	Healthy control; not a diabetic non-DR comparator
GSM5693109	Control - Clustering group A - #5	Excluded	No	Healthy control; not a diabetic non-DR comparator
GSM5693110	Control - Clustering group A - #6	Excluded	No	Healthy control; not a diabetic non-DR comparator
GSM5693111	Control - Clustering group A - #7	Excluded	No	Healthy control; not a diabetic non-DR comparator
GSM5693112	Control - Clustering group A - #8	Excluded	No	Healthy control; not a diabetic non-DR comparator
GSM5693113	Control - Clustering group A - #9	Excluded	No	Healthy control; not a diabetic non-DR comparator
GSM5693114	Type 2 diabetes without complications within 5 years - clustering group B - #1	DM no DR / T2DwtC	Yes	Included as diabetic non-DR comparator
GSM5693115	Type 2 diabetes without complications within 5 years - clustering group B - #2	DM no DR / T2DwtC	Yes	Included as diabetic non-DR comparator
GSM5693116	Type 2 diabetes without complications within 5 years - clustering group B - #3	DM no DR / T2DwtC	Yes	Included as diabetic non-DR comparator
GSM5693117	Type 2 diabetes without complications within 5 years - clustering group B - #4	DM no DR / T2DwtC	Yes	Included as diabetic non-DR comparator
GSM5693118	Type 2 diabetes without complications within 5 years - clustering group C - #1	DM no DR / T2DwtC	Yes	Included as diabetic non-DR comparator
GSM5693119	Type 2 diabetes without complications within 5 years - clustering group C - #2	DM no DR / T2DwtC	Yes	Included as diabetic non-DR comparator
GSM5693120	Type 2 diabetes without complications within 5 years - clustering group C - #3	DM no DR / T2DwtC	Yes	Included as diabetic non-DR comparator
GSM5693121	Type 2 diabetes without complications within 5	DM no DR / T2DwtC	Yes	Included as diabetic non-DR comparator

	years - clustering group C - #4			
GSM5693122	Type 2 diabetes without complications within 5 years - clustering group C - #5	DM no DR / T2DwtC	Yes	Included as diabetic non-DR comparator
GSM5693123	Type 2 diabetic nephropathy within 5 years - clustering group B - #1	Excluded	No	T2DN; excluded from DR versus diabetic non-DR comparison
GSM5693124	Type 2 diabetic nephropathy within 5 years - clustering group B - #2	Excluded	No	T2DN; excluded from DR versus diabetic non-DR comparison
GSM5693125	Type 2 diabetic retinopathy within 5 years - clustering group B - #1	T2DR	Yes	Included as DR case
GSM5693126	Type 2 diabetic retinopathy within 5 years - clustering group B - #2	T2DR	Yes	Included as DR case
GSM5693127	Type 2 diabetic retinopathy within 5 years - clustering group C - #1	T2DR	Yes	Included as DR case
GSM5693128	Type 2 diabetic retinopathy within 5 years - clustering group C - #2	T2DR	Yes	Included as DR case
GSM5693129	Type 2 diabetic nephropathy within 5 years - clustering group C - #1	Excluded	No	T2DN; excluded from DR versus diabetic non-DR comparison
GSM5693130	Type 2 diabetic nephropathy within 5 years - clustering group C - #2	Excluded	No	T2DN; excluded from DR versus diabetic non-DR comparison
GSM5693131	Type 2 diabetic retinopathy within 5 years - clustering group C - #3	T2DR	Yes	Included as DR case
GSM5693132	Type 2 diabetes without complications above 15 years - clustering group B - #1	DM no DR / T2DwtC	Yes	Included as diabetic non-DR comparator
GSM5693133	Type 2 diabetes without complications above 15 years - clustering group B - #2	DM no DR / T2DwtC	Yes	Included as diabetic non-DR comparator
GSM5693134	Type 2 diabetes without complications above 15 years - clustering group B - #3	DM no DR / T2DwtC	Yes	Included as diabetic non-DR comparator
GSM5693135	Type 2 diabetes without complications above 15 years - clustering group B - #4	DM no DR / T2DwtC	Yes	Included as diabetic non-DR comparator
GSM5693136	Type 2 diabetes without complications above 15 years - clustering group C - #1	DM no DR / T2DwtC	Yes	Included as diabetic non-DR comparator
GSM5693137	Type 2 diabetes without complications above 15 years - clustering group C - #2	DM no DR / T2DwtC	Yes	Included as diabetic non-DR comparator

GSM5693138	Type 2 diabetes without complications above 15 years - clustering group C - #3	DM no DR / T2DwtC	Yes	Included as diabetic non-DR comparator
GSM5693139	Type 2 diabetes without complications above 15 years - clustering group C - #4	DM no DR / T2DwtC	Yes	Included as diabetic non-DR comparator
GSM5693140	Type 2 diabetes without complications above 15 years - clustering group C - #5	DM no DR / T2DwtC	Yes	Included as diabetic non-DR comparator
GSM5693141	Type 2 diabetic nephropathy above 15 years - clustering group B - #1	Excluded	No	T2DN; excluded from DR versus diabetic non-DR comparison
GSM5693142	Type 2 diabetic nephropathy above 15 years - clustering group B - #2	Excluded	No	T2DN; excluded from DR versus diabetic non-DR comparison
GSM5693143	Type 2 diabetic retinopathy above 15 years - clustering group B - #1	T2DR	Yes	Included as DR case
GSM5693144	Type 2 diabetic retinopathy above 15 years - clustering group B - #2	T2DR	Yes	Included as DR case
GSM5693145	Type 2 diabetic retinopathy above 15 years - clustering group C - #1	T2DR	Yes	Included as DR case
GSM5693146	Type 2 diabetic retinopathy above 15 years - clustering group C - #2	T2DR	Yes	Included as DR case
GSM5693147	Type 2 diabetic nephropathy above 15 years - clustering group C - #1	Excluded	No	T2DN; excluded from DR versus diabetic non-DR comparison
GSM5693148	Type 2 diabetic nephropathy above 15 years - clustering group C - #2	Excluded	No	T2DN; excluded from DR versus diabetic non-DR comparison
GSM5693149	Type 2 diabetic retinopathy above 15 years - clustering group C - #3	T2DR	Yes	Included as DR case
